# Increased Levels of IgG Antibodies against Human HSP60 in Patients with Spondyloarthritis

**DOI:** 10.1371/journal.pone.0056210

**Published:** 2013-02-12

**Authors:** Astrid Hjelholt, Thomas Carlsen, Bent Deleuran, Anne Grethe Jurik, Berit Schiøttz-Christensen, Gunna Christiansen, Svend Birkelund

**Affiliations:** 1 Department of Biomedicine – Medical Microbiology and Immunology, Aarhus University, Aarhus, Denmark; 2 Department of Health Science and Technology, Aalborg University, Aalborg, Denmark; 3 Department of Rheumatology, Aarhus University Hospital, Aarhus, Denmark; 4 Department of Radiology, Aarhus University Hospital, Aarhus, Denmark; 5 Aarhus Clinic for Rheumatic Diseases, Aarhus, Denmark; 6 Loke Diagnostics, Risskov, Denmark; Albert Einstein Institute for Research and Education, Brazil

## Abstract

Spondyloarthritis (SpA) comprises a heterogeneous group of inflammatory diseases, with strong association to human leukocyte antigen (HLA)-B27. A triggering bacterial infection has been considered as the cause of SpA, and bacterial heat shock protein (HSP) seems to be a strong T cell antigen. Since bacterial and human HSP60, also named HSPD1, are highly homologous, cross-reactivity has been suggested in disease initiation. In this study, levels of antibodies against bacterial and human HSP60 were analysed in SpA patients and healthy controls, and the association between such antibodies and disease severity in relation to HLA-B27 was evaluated.

Serum samples from 82 patients and 50 controls were analysed by enzyme-linked immunosorbent assay (ELISA) for immunoglobulin (Ig)G1, IgG2, IgG3 and IgG4 antibodies against human HSP60 and HSP60 from *Chlamydia trachomatis, Salmonella enteritidis* and *Campylobacter jejuni*. Disease severity was assessed by the clinical scorings Bath Ankylosing Spondylitis Disease Activity Index (BASDAI), Bath Ankylosing Spondylitis Functional Index (BASFI) and Bath Ankylosing Spondylitis Metrology Index (BASMI).

Levels of IgG1 and IgG3 antibodies against human HSP60, but not antibodies against bacterial HSP60, were elevated in the SpA group compared with the control group. Association between IgG3 antibodies against human HSP60 and BASMI was shown in HLA-B27^+^ patients. Only weak correlation between antibodies against bacterial and human HSP60 was seen, and there was no indication of cross-reaction.

These results suggest that antibodies against human HSP60 is associated with SpA, however, the theory that antibodies against human HSP60 is a specific part of the aetiology, through cross-reaction to bacterial HSP60, cannot be supported by results from this study. We suggest that the association between elevated levels of antibodies against human HSP60 and disease may reflect a general activation of the immune system and an increased expression of human HSP60 in the synovium of patients with SpA.

## Introduction

Spondyloarthritis (SpA) comprises a heterogeneous group of chronic inflammatory diseases, including reactive arthritis, ankylosing spondylitis, psoriatic arthritis, inflammatory bowel disease (IBD) associated arthritis and undifferentiated spondyloarthritis. The clinical picture of these diseases is similar and characterized by inflammation of joints and entheses, predominantly in the spine and pelvis [1]. Assessments of disease activity, the physical function and the spinal mobility of ankylosing spondylitis patients are performed by the Bath Ankylosing Spondylitis Disease Activity Index (BASDAI), the Bath Ankylosing Spondylitis Functional Index (BASFI) and the Bath Ankylosing Spondylitis Metrology Index (BASMI), respectively. These indexes are based on clinical measurements (BASMI) along with questions pertaining degree of functional limitation and symptoms (BASDAI and BASFI) [2]. In this study BASDAI, BASFI and BASMI are considered parameters of experienced disease severity.

The pathogenesis of SpA is not fully elucidated, however, there is a remarkable hereditary component, and several MHC class I genes have been linked to disease. Best characterized is the association with human leukocyte antigen (HLA)-B27. The frequency of HLA-B27 varies in different spondyloarthritides [3]. The mechanisms underlying the influence of HLA-B27 on disease susceptibility are poorly understood. However, an interaction between HLA-B27 and a triggering bacterium as part of disease initiation has been suggested, partly based on animal studies [1], [3]. In such a study HLA-B27 transgenic rats developed spondyloarthritis-like features, only if bred in a non-germ-free environment [4]. Moreover, reactive arthritis is in humans triggered by infection with urogenital or gastrointestinal bacteria such as *Salmonella enterica*, *Campylobacter jejuni*, *Yersinia enterocolitica* and *Chlamydia trachomatis* [5]. However, evidence supporting a preceding bacterial infection in other spondyloarthritides is limited [1], and the potential interplay between HLA-B27 and bacteria in the development of SpA in humans remains to be proven.

Bacterial heat shock protein (HSP) 60 has been shown to generate a strong immune response in patients suffering from reactive arthritis [6], [7]. In humans, HSP60 is also named HSPD1 [8]. HSPs are a group of functionally related chaperones found in both eukaryotic and prokaryotic organisms. They are stress-proteins, and their expression is significantly up-regulated when cells are exposed to stressors, such as increased temperature, oxidative stress or inflammation [9]. Over the last few years it has been established that in addition to their function as intracellular chaperones, HSPs are also found in the cell membrane and outside the cell, presumably acting as indicators of the stressful conditions, activating other cells, particularly cells of the immune system [10]. It is assumed that circulating HSPs can have immunostimulating as well as immunosuppressing effects, depending on the circumstances by which they interact with other cells [10].

HSPs are highly conserved throughout evolution with considerable sequence homology between various species, and cross-reactivity between bacterial and human HSP60 has been connected with a number of inflammatory disorders [11], [12]. Rheumatologic studies have primarily focused on the role of HSP60 in rheumatoid arthritis [13], whereas less studies are done on SpA. However, autoimmunity against HSP60 has also been suggested as a part of the aetiology in this group of diseases [14].

The aim of this study was to analyse serum levels of antibodies against bacterial and human HSP60 in SpA patients compared with an age and gender matched control group. Though other studies have examined the association between bacterial HSP60 and SpA [15], [16], [17], only one previous study has measured antibodies to human HSP60 in SpA patients [17]. Previous studies measuring IgG against HSP60 did not determine subclass specificity. However, since the significance of different IgG subclasses in relation to other inflammatory diseases has been described [18], this study evaluates the IgG subclasses to bacterial and human HSP60. In addition to the analyses of antibody levels in SpA patients compared with healthy controls, the association between antibody levels and disease severity assessed by the disease parameters BASDAI, BASFI and BASMI in relation to HLA-B27 status was evaluated.

## Materials and Methods

### Serum samples

Serum samples from SpA patients with symptoms restricted to the axial skeleton (n = 82) were obtained, together with HLA-B27 status and the clinical scorings, BASDAI, BASFI and BASMI. All patients met the European Spondyloarthropathy Study Group (ESSG) criteria [19]. Serum from age and gender matched normal healthy volunteers (n = 50) served as controls (control group) (Table 1).

**Table 1:** Characteristics of SpA patients, analysed in total and divided in the HLA-B27^+^- and the HLA-B27^−^-group, and healthy controls.

Characteristics	SpA patients (n = 82)			Controls (n = 50)
	Total (n = 82)	HLA-B27^+^ (n = 47)	HLA-B27^−^ (n = 30)	
**Age, years, mean (CI)**	37 (35;39)	36 (34;39)	37 (34;41)	38 (21;65)
**Gender, females, no. (%)**	55 (57)	22 (47)	22 (73)	28 (56)
**Disease duration, years, mean (CI)**	8 (7;9)	9 (7;10)	7 (6;8)	NA
**Treatment**				
**None, no. (%)**	64 (78)	36 (77)	24 (80)	NA
**Methotrexate, no. (%)**	8 (10)	4 (9)	3 (10)	NA
**Salazopyrin, no. (%)**	9 (11)	6 (13)	3 (10)	NA
**Anti-TNF, no. (%)**	6 (7)	5 (11)	0 (0)	NA
**CRP mg/L (CI)**	2.2 (1.5;3.9)	5.5 (2.2;9.1)	2.1 (1.5;2.7)	-
**BASDAI, mean (CI)**	32 (27;37)	27 (20;34)	39 (30;49)	NA
**BASMI, mean (CI)**	5 (2;8)	8 (3;12)	1 (0;3)	NA
**BASFI, mean (CI)**	20 (16;25)	17 (11;23)	25 (16;34)	NA

*SpA* spondyloarthritis, *CRP* C-reactive protein, *BASDAI* Bath Ankylosing Spondylitis Disease Activity Index, *BASMI* Bath Ankylosing Spondylitis Metrology index, *BASFI* Bath Ankylosing Functional index, *CI* confidence interval.

The patients were enrolled in the study and serum was collected from the outpatient clinic at Aarhus University Hospital after informed written consent was given, according to the Danish Data Protection Agency, the Local Ethics Committee (project number 20050046) and the Declaration of Helsinki.

### Characteristics of the patients

Characteristics of the patient group are shown in Table 1. The age and gender of the patient and control group were comparable. As expected, the number of HLA-B27 positive persons was higher in the patient group (57%) than in the control group (8% in Caucasians) [20]. The average disease duration was eight years. Most of the patients did not receive any treatment at the time of enrolment in the study in agreement with their CRP being within the normal range (Table 1).

### Enzyme-linked immunosorbent assay (ELISA)

Prevalence of antibodies was determined by enzyme-linked immunosorbent assay (ELISA) using IgG subclass-specific secondary antibodies.

*cHSP60-IgG-ELISA* plates (Medac, Hamburg, Germany) [21] were used for *C. trachomatis* HSP60. The ELISA for *Campylobacter jejuni*, *Salmonella enteritidis* and human HSP60 were performed as described [22]. ELISA plates were coated with 4 µg/ml human HSP60, *C. jejuni* HSP60 or *S. enteritidis* HSP60.

Full length human HSP60 was obtained from Loke Diagnostics (Risskov, Denmark). *C. jejuni* and *S. enteritidis* HSP60 genes were cloned in pET30ek-LIC vector (Invitrogen, Carlsbad, CA, USA). The *C. jejuni* HSP60 gene was amplified with the forward primer 5′GACGACGACAAGATGGCAAAAGAAATTATTTTTTCAGATGAAGC3′ and reverse primer 5′GAGGAGAAGCCCGGTTTACATCATTCCTCCCATGCC3′. For *S. enteritidis* HSP60 gene, the primers 5′GACGACGACAAGATGGCAGCTAAAGACGTAAAATTCGG3′ and 5′GAGGAGAAGCCCGGTTTACATCATGCCGCCC3′ were used. The PCR products were cloned into pET30ek-LIC by ligase independent cloning, according to the manufacturer's instructions. The proteins were expressed in *Escherichia coli* BL21 (DE3) using 1 mM isopropyl-β-D-thio-galactoside (IPTG) for two hrs. The recombinant HSP60 proteins were purified by Ni^2+^ affinity chromatography under native conditions according to Schmitt et al. (1993) [23].

The human sera were diluted 1∶50 in Bac-dil (Medac) before use. The secondary anti-human IgG antibodies used were horseradish peroxidase (HRP) conjugated, sheep-anti-human IgG1, IgG2, IgG3 and IgG4, (Binding site, Birmingham, UK), diluted 1∶10,000 in Bac-dil. The dilutions were chosen so that the OD_450 nm_ levels were within the linear part of the standard curve.

For quantification of IgG subclasses, NUNC MaxiSorp plates were coated with dilution series of native IgG1, IgG2, IgG3 and IgG4 from human myeloma plasma (EMD Biosciences, San Diego, CA, USA) in CCB-buffer (50 mM NaHCO_3_, pH 9.6). The respective secondary antibodies were added to the dilutions.

In this study, inter-assay and intra-assay variability were less than 10% and 5%, respectively.

### Statistical analysis

The data were analysed by GraphPad Prism version 5.0a for Mac OS X (Graphpad Software Inc., La Jolla, CA, USA), using individual samples as experimental unit. Mann-Whitney U-test was used to analyse the differences between antibody levels in the two groups (SpA and control group) and between IgG1 and IgG3 antibody levels. Spearman nonparametric correlation was used to analyse the correlation between antibody levels, and between antibody levels and the disease parameters BASDAI, BASFI and BASMI. Probabilities <0.01 were considered as significant. The detection limits were calculated as the standard deviations (SD) of the blanks (wells incubated without sample) times two [cut-off = SD (Blanks) * 2].

## Results

### Antibodies against bacterial and human HSP60 in SpA patients and healthy controls

**Figure 1:**
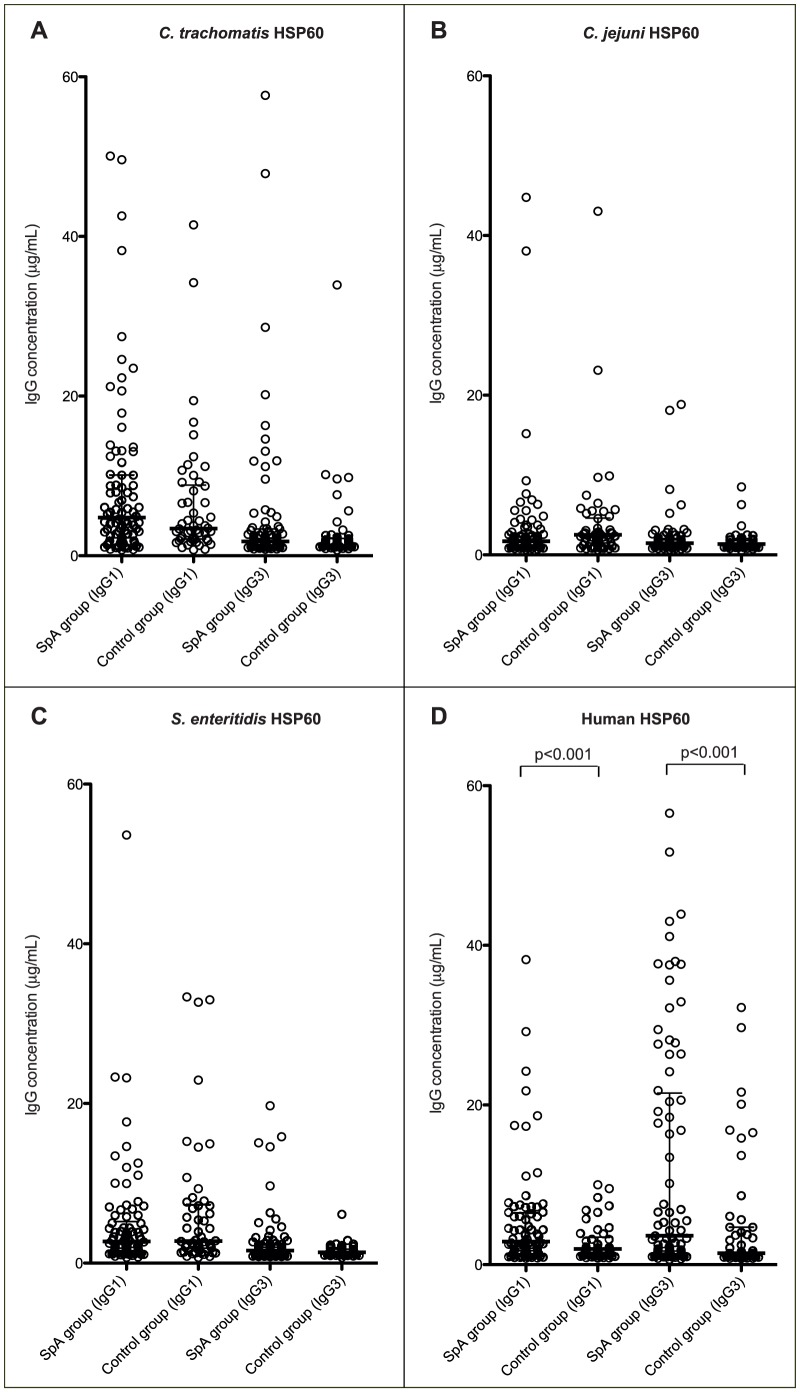
Antibody levels in the SpA group and the control group. Serum levels (µg/mL) of IgG1 and IgG3 antibodies against *C. trachomatis* HSP60 (A), *C. jejuni* HSP60 (B), *S. enteritidis* HSP60 (C) and human HSP60 (D) in the SpA group and the control group. No differences in serum levels of antibodies against the bacterial HSP60 were found between the two groups (A–C). Levels of anti-human HSP60 IgG1 and IgG3 were elevated in the SpA group compared with the control group (D). The two groups are compared using the non-parametric Mann-Whitney rank sum test. Bars represent medians with interquartile ranges (IQR).

The levels of antibodies against bacterial HSP60 in the SpA group did not differ from the control group (Figure 1A, 1B, 1C). IgG1 and IgG3 antibodies against HSP60 from all three bacteria were frequently detected in both groups. The level of IgG1 was significantly higher than the IgG3 level (Figure 1A, 1B, 1C). Medians and interquartile ranges (IQR) of IgG1 and IgG3 antibody levels (µg/mL) against human and bacterial HSP60 in the SpA group are shown in Table 2.

**Table 2:** Medians and interquartile ranges (IQR) of IgG1 and IgG3 antibody levels (µg/mL) against human and bacterial HSP60 in the SpA group.

	Median (IQR)		Statistical result of the Mann Whitney U test (p-value)
	IgG1	IgG3	
Human HSP60	2.91 (1.71;6.53)	3.38 (1.31;21.42)	P = 0.53
*C. trachomatis* HSP60	5.50 (3.07;10.81)	1.52 (1.07;3.08)	p<0.0001
*C. jejuni* HSP60	2.14 (1.64;3.22)	1.64 (1.41;2.08)	P = 0.0001
*S. enteritidis* HSP60	3.22 (2.00;6.03)	1.55 (1.12;2.31)	p<0.0001

The two groups are compared using the non-parametric Mann-Whitney rank sum test.

IgG2 antibodies were only demonstrated against HSP60 from *S. enteritidis* and *C. jejuni* in a few SpA patients (n = 5 and n = 1, respectively, data not shown). IgG4 antibodies were below detection limit (0.03 µg/ml) in all individuals.

IgG antibodies against human HSP60 were detected in both SpA patients and healthy controls. However, serum levels of both IgG1 and IgG3 antibodies against human HSP60 were higher in the SpA group compared with the control group (p<0.0001, Figure 1D). As demonstrated for bacterial HSP60, the IgG subclasses produced against human HSP60 were IgG1 and IgG3, whereas no IgG2 and IgG4 were detected (detection limit 0.03 µg/ml). There was no significant difference between levels of IgG1 and IgG3, however a trend towards a higher IgG3 level was observed (Figure 1D, Table 2). There was no correlation between serum levels of IgG1 and IgG3 against human HSP60.

Weak correlations between IgG1 antibodies against human HSP60 and IgG1 against HSP60 from the three bacteria species were found, whereas there was no correlation between IgG3 against human and bacterial HSP60 (Table 3). Also, correlation between IgG1 against the three bacterial HSP60, as well as IgG3 against the three bacterial HSP60, was observed (data not shown). The strongest correlation was between IgG1 against HSP60 from *C. trachomatis* and *C. jejuni* (r = 0.49, p<0.0001, data not shown).

**Table 3:** The Spearman rank correlation coefficient, r, for the correlation between levels of IgG1 and IgG3 against bacterial and human HSP60.

	*C. trachomatis* (IgG1)	*C. trachomatis* (IgG3)	*S. enteritidis* (IgG1)	*S. enteritidis* (IgG3)	*C. jejuni* (IgG1)	*C. jejuni* (IgG3)
Human (IgG1)	0.32**	0.01	0.26*	0.06	0.26*	0.01
Human (IgG3)	0.02	0.12	−0.02	0.19	−0.06	0.12

** p<0.001;

* p<0.01.

No association was found between antibody levels and the patients' age, sex or treatment.

### Association between antibody levels, HLA-B27 status and the clinical parameters BASDAI, BASFI and BASMI

IgG3, but not IgG1, antibodies against human HSP60 were correlated with BASMI (Spearman r = 0.34, p = 0.003, Table 4), whereas no connection was found to BASDAI and BASFI. There was no association between antibodies against bacterial HSP60 and BASDAI, BASFI, or BASMI.

**Table 4:** The Spearman rank correlation coefficient, r, for the correlation between levels of IgG1 and IgG3 against human HSP60 and BASDAI, BASMI and BASMI in the total group of SpA patients, the group of HLA-B27 positive patients (HLA-B27^+^) and the group of HLA-B27 negative patients (HLA-B27^−^).

	Total SpA group		HLA-B27^+^		HLA-B27^−^	
	IgG1	IgG3	IgG1	IgG3	IgG1	IgG3
**BASDAI**	0.13	0.05	0.18	0.05	0.05	0.02
**BASFI**	−0.03	0.01	0.08	0.00	−0.27	−0.13
**BASMI**	0.16	0.34*	0.29	0.48**	−0.13	0.05

** p<0.001;

* p<0.01.

Patients were divided into two groups based on HLA-B27 status (HLA-B27^+^, n = 47; HLA-B27^−^, n = 30, Table 4). There was no difference in antibody levels in the two groups (Figure S1). A larger part of the patients in the HLA-B27^−^ group had a BASMI score of zero compared with the patients in the HLA-B27^+^ group, and a tendency towards a higher mean BASMI score was seen in the HLA-B27^+^ group compared with the HLA-B27^−^ group (p = 0.02). Contrary to this, the mean value of BASFI (p = 0.13) and BASDAI (p = 0.05) tended to be higher in the HLA-B27^−^ group (Table 1a). However, no significant differences in BASDAI, BASFI and BASMI scores were observed between the two groups (Table 1). In the HLA-B27^+^ group the correlation between IgG3 against human HSP60 and BASMI was recognized again, and the association was stronger than observed in the total group of patients (Spearman r = 0.48, p = 0.001, Table 4). There was no correlation to bacterial HSP60. In the HLA-B27^−^ group, no correlations were observed to neither bacterial nor human HSP60.

## Discussion

In this study, elevated levels of antibodies to human HSP60 but not bacterial HSP60 were found in patients with SpA. Correlation between antibodies against HSP60 from different bacteria was demonstrated, but no evidence of cross-reaction to human HSP60 was found, since only limited correlation occurred between antibodies to human and bacterial HSP60. Furthermore, the predominant IgG subclasses produced in response to human and bacterial HSP60 were different, IgG3 and IgG1, respectively.

The connection between antibodies against human HSP60 and SpA, reflected by the elevated antibody levels, was further supported by the fact that also the disease severity assessed by the clinical disease index, BASMI, was positively associated with antibodies against human HSP60. This association indicates that a higher antibody level against human HSP60 is connected to more severe disease. BASMI evaluates the mobility of the spine and more severe disease is therefore likely to reflect a higher degree of inflammation in joints and entheses. Altogether, this suggests that the level of antibodies against human HSP60 is related to the degree of inflammation.

The analysis of the HLA-B27^+^- and the HLA-B27^−^-group showed that the association between antibodies to human HSP60 and BASMI was restricted to HLA-B27 positive patients, whereas no correlation was seen in the HLA-B27^−^ group. No difference in antibody levels between the two groups was evident, demonstrating that HLA-B27 is not essential in the generation of antibodies against human HSP60. However, the results suggest that the HLA-B27 allele may facilitate the association between antibodies to human HSP60 and joint inflammation. This is possibly reflecting a difference in the pathogenesis between the SpA diseases, since the frequency of HLA-B27 varies in the different diseases comprising the SpA group [3]. For example, in ankylosing spondylitis 96% of patients are HLA-B27 positive [25], whereas in IBD associated arthritis the frequency of the HLA-B27 allele is 40–60% [26], [27]. Consequently, among HLA-B27 positive patients, ankylosing spondylitis is likely to be overrepresented compared with IBD associated arthritis. The association to the HLA-B27 allele may, however, be explained by the fact that a larger part of the HLA-B27 negative patients had a BASMI score of zero, compared with the HLA-B27 positive patients, though no significant difference between the two groups was evident.

A potential association between HLA-B27 and generation of antibodies is supported by findings from previous studies reporting an increased level of antibodies to bacterial HSP60 in HLA-B27 positive individuals [15], [16]. Many hypothesises regarding the correlation to HLA-B27 have been suggested, including molecular mimicry, the arthritogenic peptide hypothesis, which postulates that HLA molecules act as a peptide-binding molecule for infectious agents, or that HLA-B27 may simply represent a marker locus, closely linked to a yet unidentified true immune response gene responsible for the inflammatory response [3]. Previous published results are ambiguous. In one study, association between antibodies to HSP60 from yersinia enterocolitica in patients suffering from uveitis was found primarily in HLA-B27 positive patients [28], whereas in another study, no difference in levels of IgA antibodies against *C. pneumoniae* HSP60 between HLA-B27 –positive and negative patients with uveitis was found [29]. In order to clarify a potential connection between HLA-B27 and the role of human HSP60, more studies, focusing on HLA-B27, need to be done.

One other study has analysed antibodies against human HSP60 in relation to a group of inflammatory diseases, including reactive arthritis [17]. This study measured total IgG against human HSP60 and reported a similar elevation of antibodies in patients with reactive arthritis. In addition, elevated levels of antibodies to *E. coli* HSP60 and positive correlation between antibodies to human and bacterial HSP60 were reported [17]. The possible link between an eliciting bacterial infection, generation of antibodies against human HSP60 and the development of SpA was not demonstrated in the present study. First, the positive correlations between IgG1 antibodies against human HSP60 and antibodies against *S. enteritidis*, *C. jejuni* and *C. trachomatis* HSP60 were weak and no correlation to IgG3 antibodies, which was the predominant subclass to human HSP60, was observed. Second, the fact that bacterial HSP60 induced an IgG1 dominated response while IgG3 was the main IgG subclass against human HSP60 makes cross-reaction unlikely. Third, even though both IgG1 and IgG3 antibodies to human HSP60 were associated with SpA, no elevation in serum levels of antibodies against HSP60 from *S. enteritidis*, *C. jejuni* and *C. trachomatis* was seen in the SpA group compared with the healthy controls. These results imply that the SpA patients, as a group, do not have a history of more frequent or severe infections with *S. enteritidis*, *C. jejuni* and *C. trachomatis*, leading to a higher antibody level, compared with the background population. This suggests that a potential causality between infection and disease is not straightforward, and that the risk of developing SpA after infection may vary between individuals. This could possibly be due to a hereditary vulnerability. However, this may also reflect the fact, that the SpA patients was analysed as a group. The group of SpA comprises a number of heterogeneous diseases, possibly with different pathogeneses and so far, only an association between reactive arthritis and bacterial infection is evident [5].

In addition, the importance of other bacteria needs to be considered. Higher levels of antibodies to HSP60 from *E. coli* and *Klebsiella pneumoniae* have been reported in patients with ankylosing spondylitis compared with a control group [15], [16]. In the present study, antibodies against HSP60 from *E. coli* and *K. pneumoniae* were not measured. The HSP60 amino acid sequences from these bacteria are almost identical to HSP60 from *S. enteritidis* (98.4% and 96.2% respectively) [30]. Consequently, the antibody epitopes are considered to be very similar, and it is not expected that measurement of antibodies against HSP60 from *E. coli* and *K. pneumoniae* would reveal a different result. HSP60 from *S. enteritidis* was chosen because at the time when most of the SpA cases developed, a high number of *S. enteritidis* infections were seen in Denmark [31]. Furthermore, *S. enteritidis* is shown to be strongly associated with reactive joints symptoms [32].

Altogether, indications supporting cross-reacting antibodies were not evident from this study. This is supported by our findings in a previous study on patients with tubal factor infertility after *C. trachomatis* infection, in which we found no indications of cross-reaction between antibodies against HSP60 from *C. trachomatis* and human HSP60 either [33].

It was evident from the present study, that the IgG antibodies produced against bacterial and human HSP60 were IgG1 and IgG3, respectively. A similar IgG subclass distribution in response to self-antigens has been demonstrated in other inflammatory diseases, including rheumatoid arthritis [18] and lupus erythematosus [34]. However, also IgG2 and IgG4 has been demonstrated to be of importance in inflammatory diseases, such as vasculitis and [18] and myasthenia gravis [35].

The main function of IgG1 and IgG3 antibodies are complement activation and opsonisation of invading microbes through FC receptor binding on macrophages. IgG3 is the strongest complement activator [36], and furthermore, IgG3 is more flexible as a result of a longer hinge region [37]. In this study, we observed a significantly higher serum level of IgG1 against bacterial HSP60 compared with the level of IgG3. Contrary, a trend towards a higher level of IgG3 compared with IgG1 antibodies against human HSP60 was observed. This is remarkable, since the general content of IgG1 in the blood is higher than IgG3 (65% and 7%, respectively) [38], [39], and the half-life of IgG3 is three times shorter than IgG1 [40]. This suggests that IgG3 is the most significant IgG subclass produced against human HSP60 in relation to SpA. Furthermore, this indicates, that IgG3 is generated continuously, possibly as a result of ongoing inflammation, whereas, IgG1 may have been generated years ago, during infection. It is possible, that the flexibility and shorter half-life of IgG3 may be of critical value for regulatory responses that can be quickly up- and down regulated.

The isotype switching and secretion of IgG subclasses are stimulated by CD4+ Th cell subsets and their cytokines [41]. However, the knowledge about the ability of specific cytokines to induce B-cells production of different IgG subclasses is controversial. This is partly because of methodological differences in the identification of B- and T cell subtypes, making comparison difficult. Furthermore, the complexity of the cytokine environment in the lymph nodes makes *in vitro* studies a rather inaccurate approximation but the inaccessibility of this B-cell compartment in humans makes *in vivo* studies difficult [42]. In studies using CD40L activated human B cells, IL-4 stimulation was predominantly associated with IgG1 expression, whereas stimulation with IL-21 primarily generated IgG3^+^ B-cells [43], indicating that these cytokines influence the IgG subclass switch. The different IgG response observed in this study may be explained by a different balance of T-cell regulatory factors at the site of antigen presentation, which is likely to occur in the joints for human HSP60, and in the gastrointestinal and urogenital tract for bacterial HSP60.

Stressed cells and tumor cells have been shown to express and release human HSP60 [44], [45]. Likewise, increased expression of human HSP60 in the synovium of patients with rheumatoid arthritis has been demonstrated [24], [46]. A similar upregulation is likely to happen in the synovium of SpA patients, which could be the explanation of the increased levels of antibodies against human HSP60 in SpA patients compared with healthy controls seen in the current study. Because of the joint inflammation related to SpA, we expect expression of human HSP60 to be increased and as a consequence, levels of antibodies may thereby be elevated. This could also explain the association between antibody levels and disease severity, demonstrated in this study. Though higher levels of antibodies and circulating HSP60 have been associated with disease, antibodies against HSP60 were also measured in the control group, just like expression of human HSP60 was found in normal synovium [46] and circulating HSP60 was detected in healthy subjects [12]. Furthermore, human HSP60 is not disease specific, but is found to be associated with a number of inflammatory diseases besides SpA. Moreover, not all patients produced antibodies against human HSP60. This indicates that an immune response against human HSP60 may not be the cause of SpA, but rather a marker reflecting inflammation. Human HSP60 is considered an immunomodulating agent, capable of interacting with the cells of the immune system [10], [47], and thereby having the potential to down- as well as up-regulate inflammation. The role of the increased expression of human HSP60 in SpA and other inflammatory diseases is still to be discovered.

In conclusion, the results of the present study support an association between SpA and a humoral immunological response towards human HSP60. No elevation of serum levels of antibodies against HSP60 from *S. enteritidis*, *C. jejuni* and *C. trachomatis* was seen in the SpA patients. Furthermore, the hypothesis regarding cross-reacting antibodies as a causal factor in SpA could not be supported. The antibody level was positively correlated with disease severity, possibly reflecting the degree of inflammation. Altogether, the theory that antibodies against human HSP60 is a specific part of the aetiology, through cross-reaction to bacterial HSP60, cannot be supported by the results from this study. However, we suggest that the association between elevated levels of antibodies against human HSP60 and disease may reflect a general activation of the immune system and an increased expression of human HSP60 in the synovium of patients with SpA.

## Supporting Information

Figure S1 **Levels of antibodies against human HSP60 in the HLA-B27^+^- and the HLA-B27^—^group.** Serum levels (µg/mL) of IgG1 and IgG3 antibodies against human HSP60 in SpA patient divided into an HLA-B27^+^- and an HLA-B27^−^-group. No difference in levels of anti-human HSP60 IgG1 and IgG3 was seen between the two groups. The groups are compared using the non-parametric Mann-Whitney rank sum test. Bars represent medians with interquartile ranges (IQR).
(EPS)
